# Addressing the need for patient-friendly medical communications: adaptation of the 2019 recommendations for the management of MPS VI and MPS IVA

**DOI:** 10.1186/s13023-022-02219-7

**Published:** 2022-03-02

**Authors:** Iain A. Bruce, Fatih S. Ezgü, Christoph Kampmann, Vladimir Kenis, William Mackenzie, Bob Stevens, Robert Walker, Christian Hendriksz

**Affiliations:** 1 Department of Paediatric Otolaryngology, Royal Manchester Children’s Hospital, Manchester Academic Health Service (MAHSC), Manchester, UK; 2 Metabolism Unit, Department of Pediatrics, Gazi University, Ankara, Turkey; 3 Division of Cardiology, University Children’s Hospital, Johannes Guterberg Universität, Mainz, Germany; 4 Department of Foot and Ankle Surgery, Neuroorthopaedics and Skeletal Dysplasias, H. Turner National Medical Research Center for Children’s Orthopedics and Trauma Surgery, Saint Petersburg, Russia; 5 Department of Orthopedics, Nemours/Alfred I, Dupont Hospital for Children, Wilmington, Delaware, USA; 6 MPS Society, Amersham, Buckinghamshire UK; 7 Department of Paediatric Anaesthesia, Royal Manchester Children’s Hospital, Manchester, UK; 8 Steve Biko Academic Unit, Department of Pediatrics, University of Pretoria, Pretoria, South Africa

**Keywords:** Rare diseases, Mucopolysaccharidosis, MPS IVA, MPS VI, Management guidelines, Enzyme replacement therapy, Surgery, Anesthetics, Patient engagement, Patient education

## Abstract

**Background:**

Patients are the most important stakeholders in the care of any disease and have an educational need to learn about their condition and the treatment they should receive. Considering this need for patient-focused materials, we present a directed approach for mucopolysaccharidosis (MPS) VI and MPS IVA, a pair of rare, inherited diseases that affects multiple organs and parts of the body. Independent guidelines on the treatment of these diseases were recently published, providing evidence- and expertise-driven recommendations to optimize patient management. However, while healthcare providers may have the training and knowledge to understand these guidelines, patients and their caregivers can find the technical content challenging. Hence, we aimed to develop plain language summaries (PLS) of the MPS VI and MPS IVA guidelines with patients as the primary audience.

**Results:**

A review of the guidelines by an expert team identified six domains of information relevant to patients: The multidisciplinary team, regular tests and check-ups, disease-modifying and supportive treatments, general anesthetics, ear-nose-throat/respiratory care, and surgeries. This information was adapted into a series of infographics specific to either MPS VI or MPS IVA, designed to appeal to patients and clearly present information in a concise manner.

**Conclusions:**

The use of patient-friendly materials, like the infographics we have developed, has the potential to better inform patients and engage them in their care. We issue a “call to arms” to the medical community for the development of similar PLS materials in rare diseases intended to inform and empower patients.

## Background

The mucopolysaccharidoses (MPS) are a varied group of rare inherited lysosomal storage disorders, in which an affected person lacks a specific enzyme that is needed to break down glycosaminoglycans (GAGs). These enzymes normally act within lysosomes, the components of the cell in which GAGs and other molecules, such as proteins, are usually broken down and recycled [1, 2]. Resulting from the lack of this enzyme, there is a progressive build-up of GAGs in organs and tissues of persons with MPS. This leads to the development of a broad range of signs and symptoms affecting different parts of the body, depending on the specific GAG and the site where they build up [3, 4]. The age when MPS becomes apparent can range from early onset (in some cases prior to birth) to late onset depending on the severity of MPS an individual has [1]. Each of the seven different types of MPS demonstrates similar but nuanced symptoms, yet each type is associated with a substantial impact on patients’ qualities of life and reduced life expectancies [3, 5].

MPS VI, also known as Maroteaux-Lamy syndrome, is caused by complete or partial loss of activity of the enzyme *N*-acetyl-galactosamine-4-sulfatase (arylsulfatase B, ARSB), which is involved in the breakdown of two different GAGs: dermatan sulfate and chondroitin 4-sulfate [6, 7]. Symptoms of MPS VI include decreased rate of bodily growth, coarse facial features, skeletal deformities, frequent upper-airway infections (e.g. influenza), hepatosplenomegaly (enlargement of the liver and spleen), hearing loss, and joint stiffness. Patients with MPS VI also have abnormalities in heart structure and function due to the build-up of dermatan sulfate within the cardiac valves [6, 7].

MPS IVA, also known as Morquio A syndrome, arises from loss of activity of the enzyme *N*-acetyl-galactosamine-6-sulfatase (GALNS), which breaks down the GAGs keratan sulfate (KS) and chondroitin sulfate (CS) [8, 9]. Accumulation of KS and CS occurs mainly in bone, cartilage, and the surrounding zone between cells called the extracellular matrix, leading to the development of skeletal deformities. Other symptoms can include respiratory (breathing) problems, snoring with breath holding (apnea), hearing loss, and dental abnormalities [8, 10]. The effects of MPS IVA on the airway can be progressive and involve more than one level within the airway, from “lips to lungs”.

As MPS affects multiple parts of the body, its management requires an extensive multidisciplinary (across multiple specialties) team of healthcare professionals (HCPs) who provide surgical, supportive, and disease-specific treatments that are tailored to each individual [7, 9]. Comprehensive management guidelines for HCPs have recently been published for MPS VI and MPS IVA, with the intention of enhancing patient quality of life and improving clinical- and patient-reported outcomes [7, 9]. These guidelines clearly highlight the importance of ensuring that patients and their caregivers are well informed of available treatment options as the diseases progress, so that they can make educated decisions to undertake any therapy and/or surgery [7, 9].

These specific guidelines demonstrate the importance of involving patients and their caregivers in healthcare decisions, and other literature has highlighted the value of engaging these groups in the planning and conduct of research so that it addresses the key evidence gaps in the management of rare diseases [11, 12]. However, despite the wide-reaching benefits of greater patient and caregiver knowledge and engagement, people living with rare diseases often have limited access to user-friendly, evidence-based information [13, 14].

To support the goal of enabling greater patient involvement in the management of their disease, scientific information needs to be more accessible, as the complex technical language often used in the medical literature can be challenging for non-scientific audiences to understand [15]. Plain language summaries (PLS), using non-technical language to describe complex medical information and concepts, have emerged as an important communication tool to enable scientific research to be more easily understood, accessible, and reach a wider audience [16].

Given the need for greater knowledge and engagement among patients with rare diseases and their caregivers, we embarked on a project to develop infographic-based PLS on the current management guidelines for MPS VI and MPS IVA [7, 9]. This article describes the methods and results of our process to produce the PLS, which aim to convey the key concepts and recommendations from the guidelines in easily understandable, patient-friendly language. We present this publication as a “call to arms” for the development of similar PLS for traditional HCP-targeted publications.

## Methods

The methods for generating the original guidelines are summarized in their respective publications [7, 9]. Briefly, both guidelines were developed using an anonymous modified Delphi method to identify guidance statements focused on key domains of care. The development of the guidelines was led by an independently selected steering committee made up of three patient advocates and 26 international HCPs from various disciplines with expertise in managing MPS VI or MPS IVA.

To adapt these guidelines, our team was assembled to include authors from multiple disciplines with specialist experience in treating patients with MPS, involving several authors of the original guidelines including a representative from a large patient advocacy organization. An initial review of the original guidelines was carried out to identify key areas of patient impact that could be developed into summaries. These summaries were then developed as visual infographics to engage a primary target audience of adolescent to young adult patients. Infographics were developed from a written outline, and then through typeset graphic design in iterations. At each step, the infographics were carefully reviewed and refined with input from the author team.

## Results

**Fig. 1:**
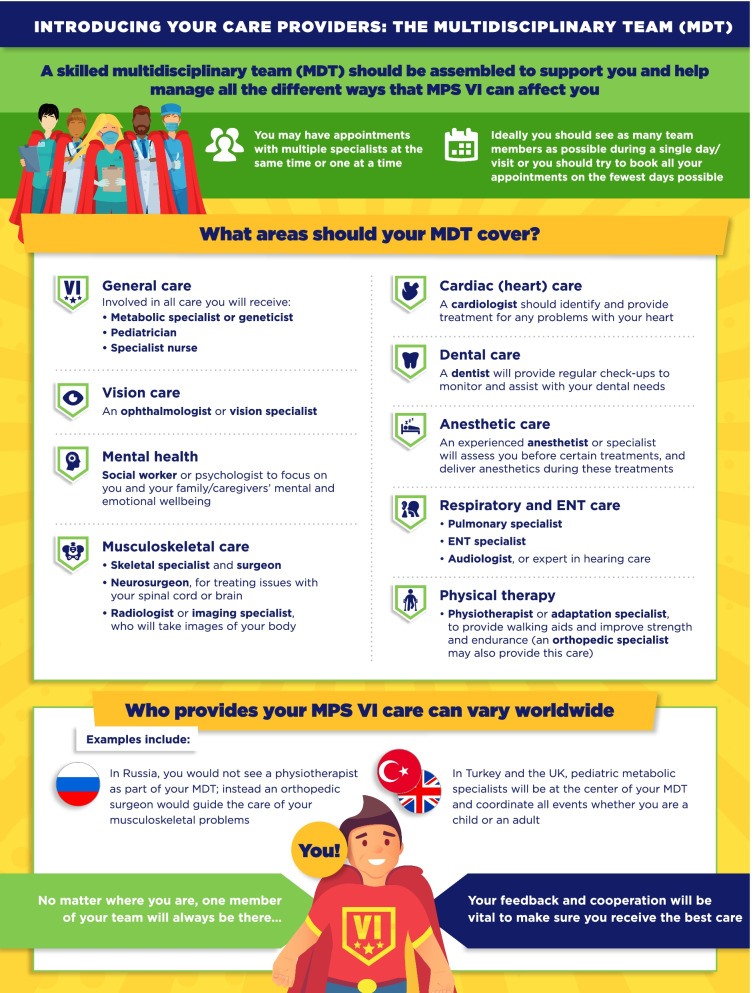
Introducing your care providers: The multidisciplinary team (MDT) in MPS VI infographic. This infographic provides a brief summary of key information found in the MPS VI treatment guidelines. For further details, please see the original paper published in the Orphanet Journal of Rare Diseases [7]. The overall Appraisal of Guidelines for Research and Evaluation (AGREE II) assessment score for the original guideline was 5.3/7 (where 1 = lowest quality and 7 = highest quality of guidance)

**Fig. 2:**
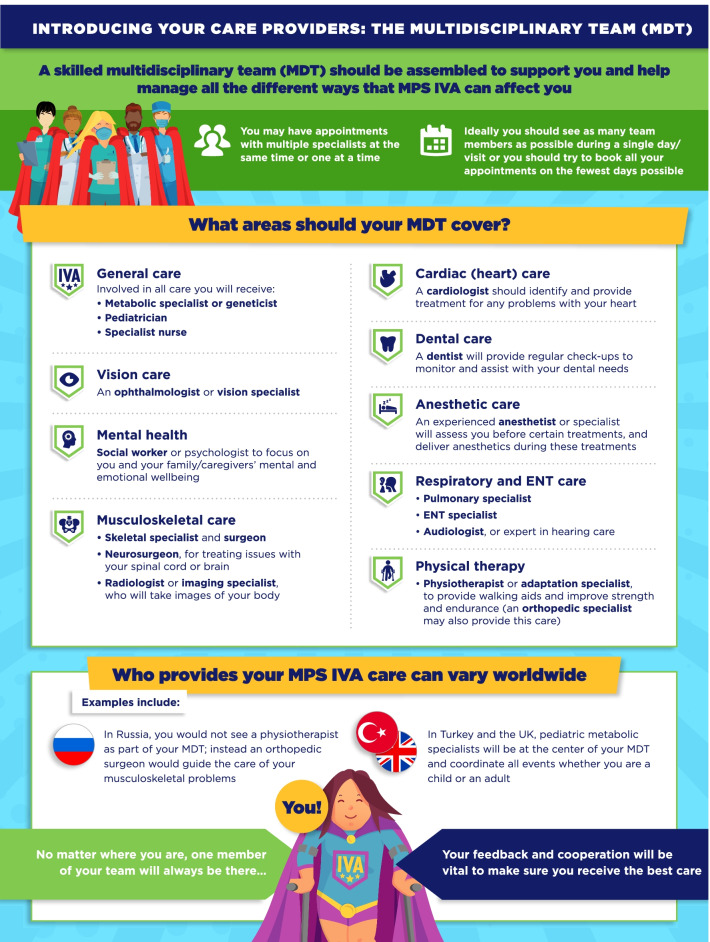
Introducing your care providers: The multidisciplinary team (MDT) in MPS IVA infographic. This infographic provides a brief summary of key information found in the MPS IVA treatment guidelines. For further details, please see the original paper published in the Orphanet Journal of Rare Diseases [9]. The overall Appraisal of Guidelines for Research and Evaluation (AGREE II) assessment score for the original guideline was 5.3/7 (where 1 = lowest quality and 7 = highest quality of guidance)

**Fig. 3:**
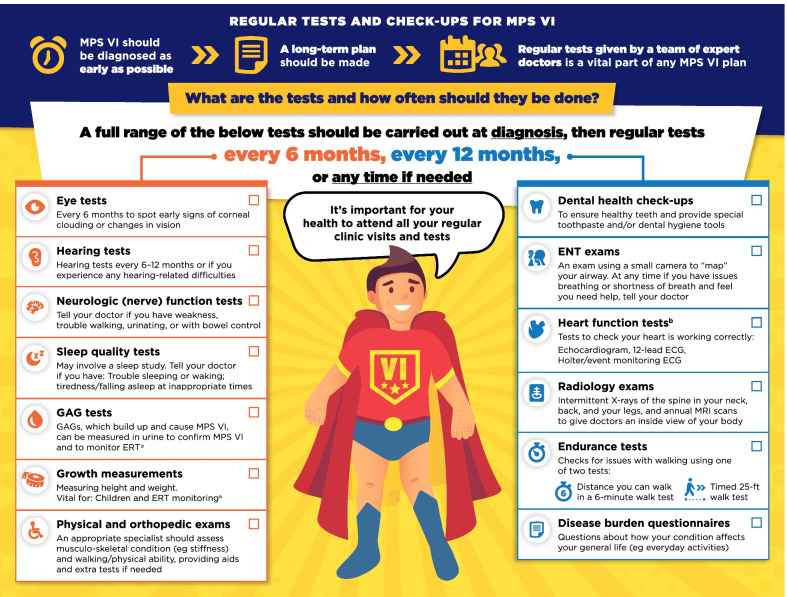
Regular tests and check-ups for MPS VI infographic. This infographic provides a summary of key information found in the MPS VI treatment guidelines. For further details, please see the original paper published in the Orphanet Journal of Rare Diseases [7]. The overall Appraisal of Guidelines for Research and Evaluation (AGREE II) assessment score for the original guideline was 5.3/7 (where 1 = lowest quality and 7 = highest quality of guidance). ^a^Please see the companion infographic "Disease-modifying and supportive treatments for MPS VI” for more information about ERT. ^b^Time between heart function tests could be longer if no issues are found

**Fig. 4:**
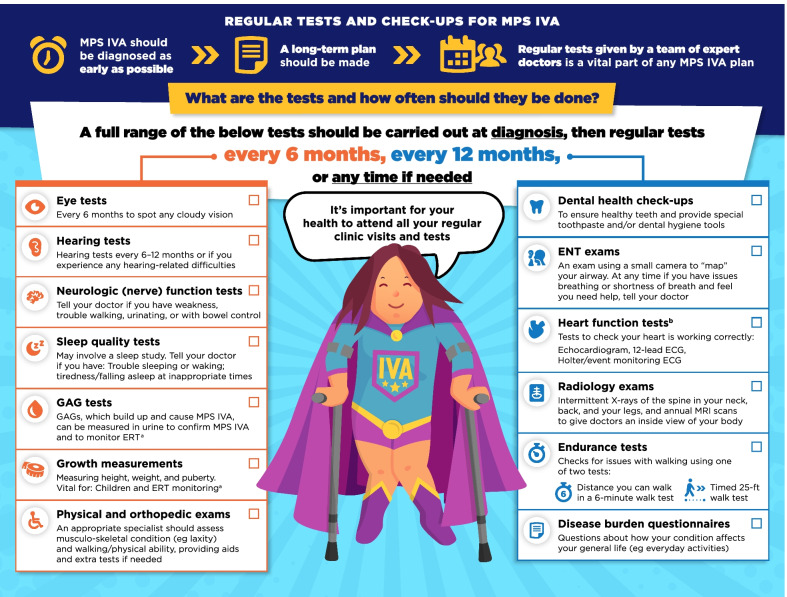
Regular tests and check-ups for MPS IVA infographic. This infographic provides a summary of key information found in the MPS IVA treatment guidelines. For further details, please see the original paper published in the Orphanet Journal of Rare Diseases [9]. The overall Appraisal of Guidelines for Research and Evaluation (AGREE II) assessment score for the original guideline was 5.3/7 (where 1 = lowest quality and 7 = highest quality of guidance). ^a^Please see the companion infographic "Disease-modifying and supportive treatments for MPS IVA” for more information about ERT. ^b^Time between heart function tests could be longer if no issues are found

**Fig. 5:**
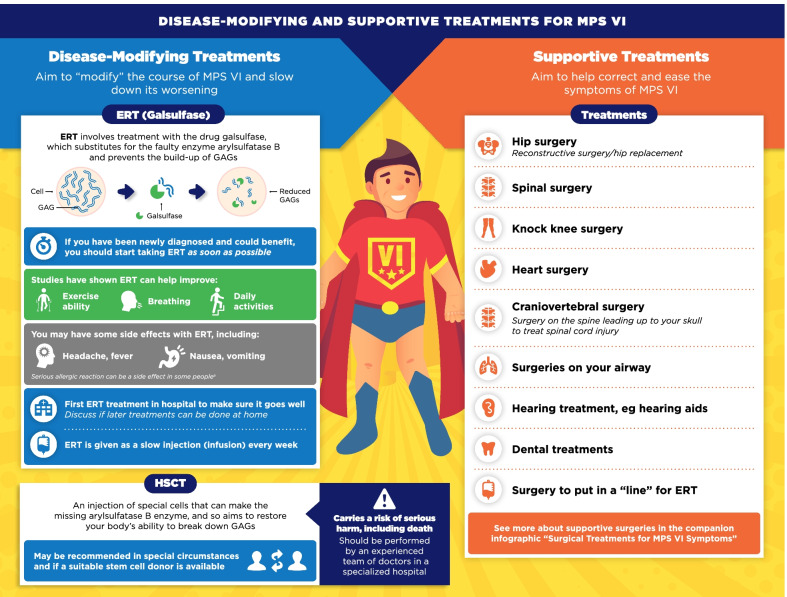
Disease-modifying and supportive treatments for MPS VI infographic. This infographic provides a summary of key information found in the MPS VI treatment guidelines. For further details, please see the original paper published in the Orphanet Journal of Rare Diseases [7]. The overall Appraisal of Guidelines for Research and Evaluation (AGREE II) assessment score for the original guideline was 5.3/7 (where 1 = lowest quality and 7 = highest quality of guidance). ^a^The prescribing information for Naglazyme (galsulfase) contains warnings for anaphylaxis and severe allergic reactions, which have been reported in clinical studies [25, 26]. Medical support should be present during all treatments. If these reactions occur, ERT infusion should be immediately halted and treatment given

**Fig. 6:**
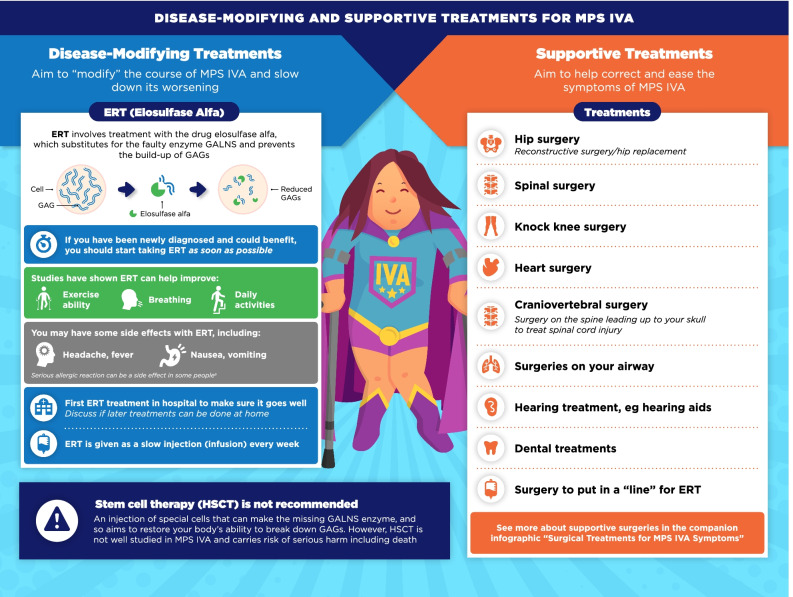
Disease-modifying and supportive treatments for MPS IVA infographic. This infographic provides a summary of key information found in the MPS IVA treatment guidelines. For further details, please see the original paper published in the Orphanet Journal of Rare Diseases [9]. The overall Appraisal of Guidelines for Research and Evaluation (AGREE II) assessment score for the original guideline was 5.3/7 (where 1 = lowest quality and 7 = highest quality of guidance). ^a^The prescribing information for Vimizim (elosulfase alfa) contains warnings for anaphylaxis and severe allergic reactions, which have been reported in clinical studies [27, 28]. Medical support should be present during all treatments. If these reactions occur, ERT infusion should be immediately halted and treatment given

**Fig. 7:**
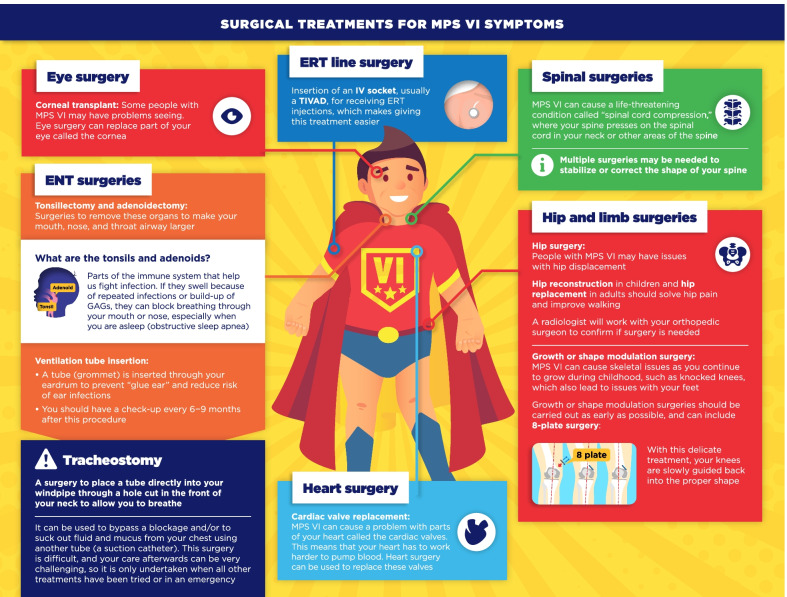
Surgical treatments for MPS VI symptoms infographic. This infographic provides a summary of key information found in the MPS VI treatment guidelines. For further details, please see the original paper published in the Orphanet Journal of Rare Diseases [7]. The overall Appraisal of Guidelines for Research and Evaluation (AGREE II) assessment score for the original guideline was 5.3/7 (where 1 = lowest quality and 7 = highest quality of guidance)

**Fig. 8:**
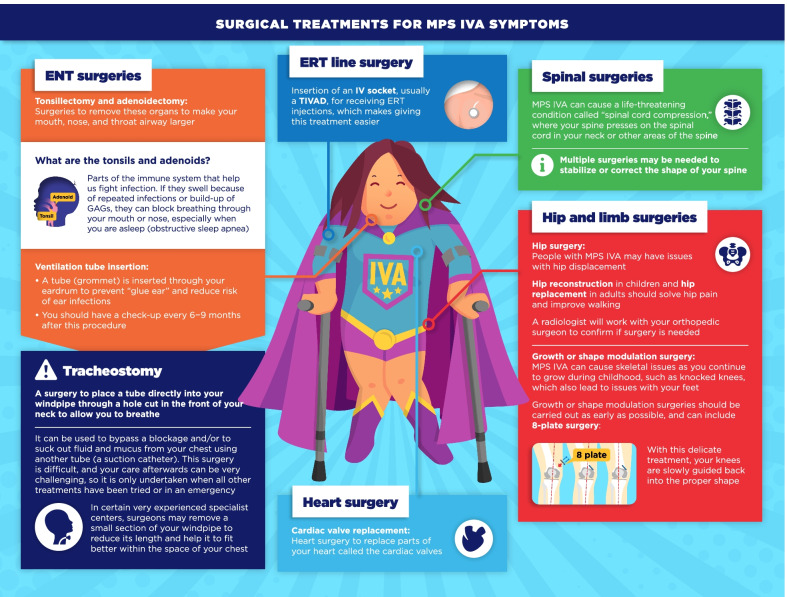
Surgical treatments for MPS IVA symptoms infographic. This infographic provides a summary of key information found in the MPS IVA treatment guidelines. For further details, please see the original paper published in the Orphanet Journal of Rare Diseases [9]. The overall Appraisal of Guidelines for Research and Evaluation (AGREE II) assessment score for the original guideline was 5.3/7 (where 1 = lowest quality and 7 = highest quality of guidance)

**Fig. 9:**
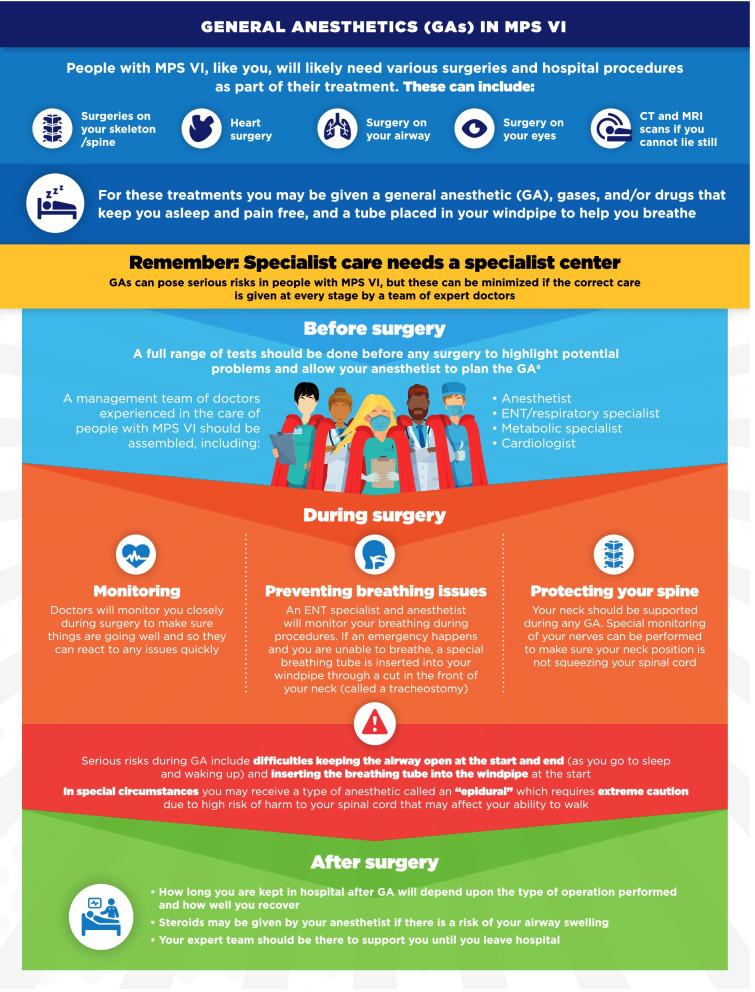
General anesthetics (GAs) in MPS VI infographic. This infographic provides a summary of key information found in the MPS VI treatment guidelines. For further details, please see the original paper published in the Orphanet Journal of Rare Diseases [7]. The overall Appraisal of Guidelines for Research and Evaluation (AGREE II) assessment score for the original guideline was 5.3/7 (where 1 = lowest quality and 7 = highest quality of guidance). ^a^For more information on the range of tests that should be carried out, please see the companion infographic “Regular tests and check-ups for MPS VI”

**Fig. 10:**
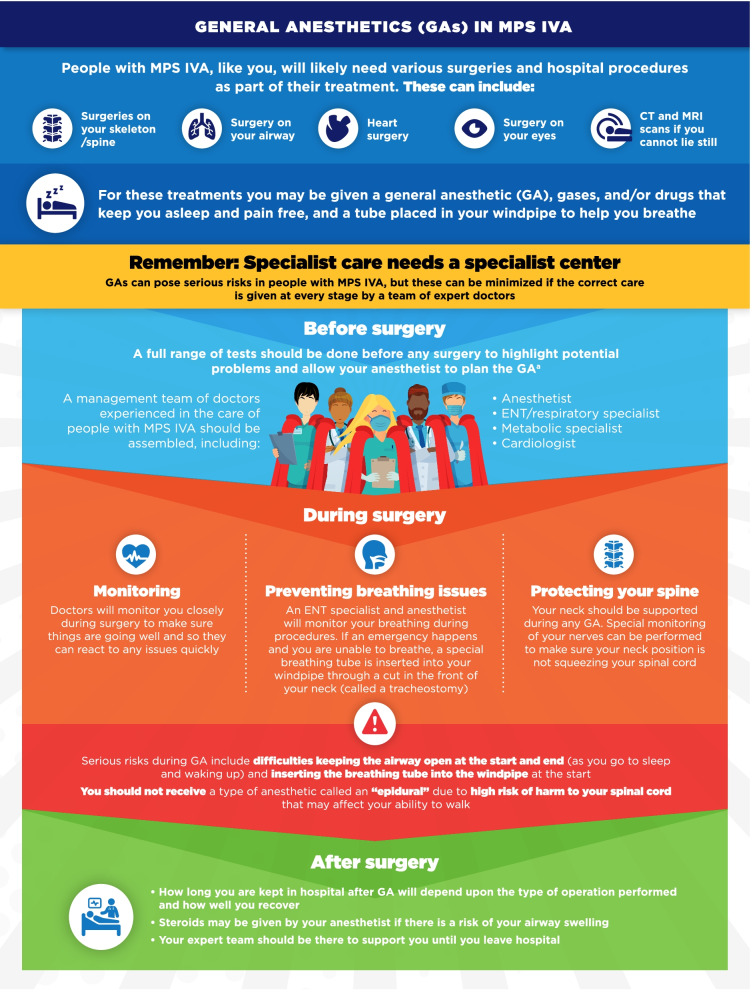
General anesthetics (GAs) in MPS IVA infographic. This infographic provides a summary of key information found in the MPS IVA treatment guidelines. For further details, please see the original paper published in the Orphanet Journal of Rare Diseases [9]. The overall Appraisal of Guidelines for Research and Evaluation (AGREE II) assessment score for the original guideline was 5.3/7 (where 1 = lowest quality and 7 = highest quality of guidance). ^a^For more information on the range of tests that should be carried out, please see the companion infographic “Regular tests and check-ups for MPS IVA”

**Fig. 11:**
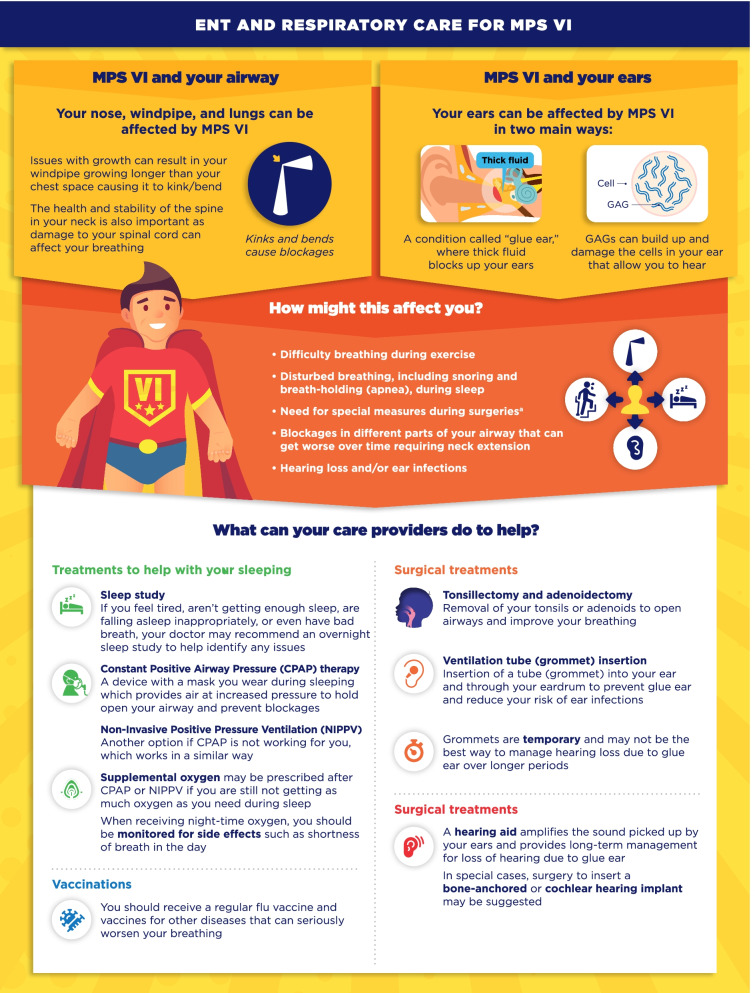
ENT and respiratory care for MPS VI infographic. This infographic provides a summary of key information found in the MPS VI treatment guidelines. For further details, please see the original paper published in the Orphanet Journal of Rare Diseases [7]. The overall Appraisal of Guidelines for Research and Evaluation (AGREE II) assessment score for the original guideline was 5.3/7 (where 1 = lowest quality and 7 = highest quality of guidance). ^a^See the companion infographic “General anesthetics for MPS VI” for more information

**Fig. 12:**
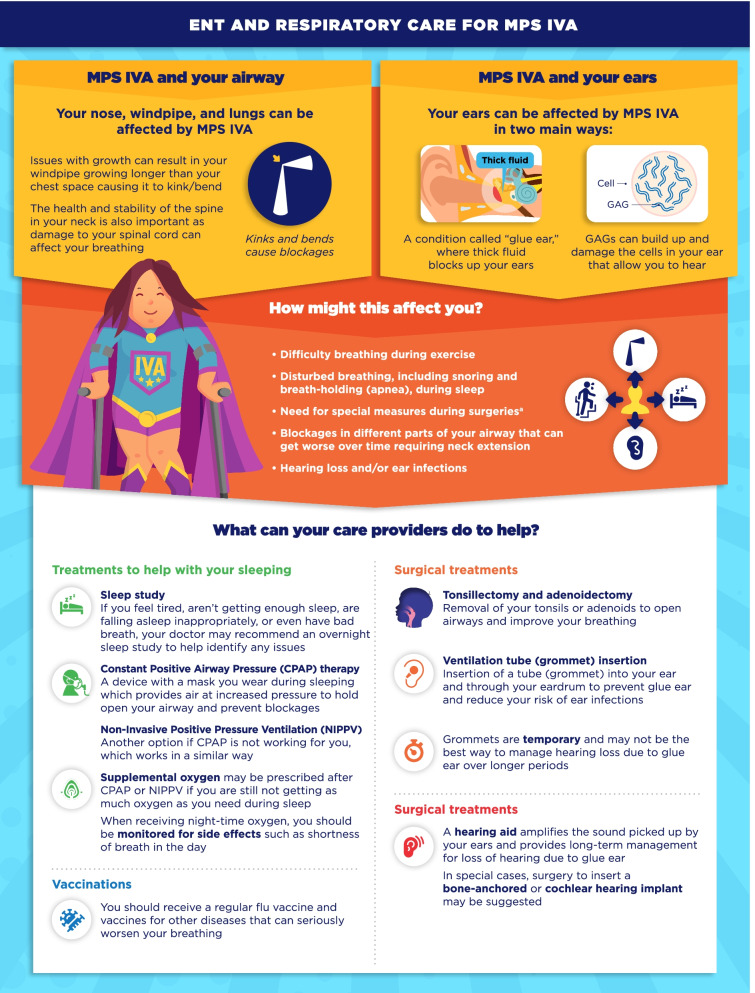
ENT and respiratory care for MPS IVA infographic. This infographic provides a summary of key information found in the MPS IVA treatment guidelines. For further details, please see the original paper published in the Orphanet Journal of Rare Diseases [9]. The overall Appraisal of Guidelines for Research and Evaluation (AGREE II) assessment score for the original guideline was 5.3/7 (where 1 = lowest quality and 7 = highest quality of guidance). ^a^See the companion infographic “General anesthetics for MPS IVA” for more information

In total, six infographic-based PLS were developed for each guideline, based on the identification of key domains with high degree of patient impact (Figs. 1, 2, 3, 4, 5, 6, 7, 8, 9, 10, 11, 12). The infographics in Figs. 1 and 2 relate to the introduction of the multidisciplinary team (MDT), which was highlighted as a core part of MPS care in both guidelines. “Introducing your care providers: The multidisciplinary team (MDT)” summarizes the MDT concept and identifies HCPs who should be involved in key areas of care, while acknowledging that the members of this team may differ depending on the healthcare system and key HCPs. It also makes clear the importance of patient involvement in decision making.

The infographics on “Regular tests and check-ups for MPS VI/IVA” adapt information from the guidelines related to routine monitoring and assessments, highlighting the schedule of key assessments and reinforcing the need for patients to attend regular clinic visits (Figs. 3, 4). To discuss current approaches to treatment and management of MPS VI and MPS IVA, the infographics “Disease-modifying and supportive treatments for MPS VI/IVA” and “Surgical treatments for MPS VI/IVA symptoms” were developed (Figs. 5, 6, 7, 8). The former discusses key information surrounding enzyme replacement therapy (ERT) and hematopoietic stem cell therapy (HSCT) in the context of MPS VI and MPS IVA, while introducing therapies aimed at addressing the symptoms of MPS. The latter infographic contains more information about select surgical interventions aimed at managing various symptoms of MPS VI and MPS IVA.

One area of key patient and physician concern regarding MPS VI and MPS IVA was identified as the use of general anesthetics (GA) for surgical and investigative procedures. The infographics on “General anesthetics (GA) in MPS VI/IVA” describe the patient journey from before, during, and after surgeries or procedures involving GA (Figs. 9, 10). Respiratory and Ear-Nose-Throat (ENT) care in MPS VI and MPS IVA is addressed in the infographics “ENT and respiratory care for MPS VI/IVA” (Figs. 11, 12). This domain describes the impact of breathing difficulties on quality of life, and the supportive care that patients can receive from their treating HCPs.

## Discussion

Despite the integral involvement of patients in the care pathway for MPS IVA and MPS VI, the complexity of existing guidelines for managing these conditions and the paucity of lay-friendly resources could represent a barrier for their engagement. Here, we have created visual infographics to relay the information from the recent specialist-targeted treatment guidelines that are most relevant to patients, and provided an outline for how patient-friendly materials may be created for other diseases. These resources may be utilized by patients but also HCPs without prior specialist knowledge of MPS diseases.

Clinical practice guidelines play an important role in educating HCPs and have been advocated as an essential part of good medical practice for several decades. When based on a systematic and critical review of the evidence, expert-led and consensus-driven guidelines can provide a powerful way to help translate the current body of knowledge into actual clinical practice, particularly in the setting of complex diseases [17, 18]. However, specialist language and the technical complexity of such guidelines may represent a barrier to non-specialists that include patients and their caregivers as well as general HCPs.

In recognition of the need to communicate this information to patient lay audiences, there has been a rapid adoption of PLS among scientific publications in recent years. The results of a survey in 2018 highlighted the value of PLS for both patients and physicians. In this survey, a major theme that emerged in patient interviews was the importance of knowledge and the sense of empowerment it brings, with patients viewing PLS as tools to enable knowledge sharing and making important information accessible. Furthermore, physicians noted the value of PLS in opening patient dialog, saving time, and streamlining communication, as their patients were not completely dependent on their doctor for information [16].

For patients and their caregivers, accurate, reliable, and up-to-date information is thus essential to equip them to make informed choices about their care; however, such information on rare diseases is often lacking. In a survey of patients and caregivers conducted in the UK covering more than 450 rare diseases, nearly 70% of respondents reported feeling that they were not provided with enough information on their condition after diagnosis, and 35% stated that they did not understand all the information they were given [19]. Similarly, in an analysis of the information needs of patients (n = 55) living with rare diseases and their relatives (n = 13) in Germany, interviewees cited a strong need for information after diagnosis on potential drug treatments and research, and a lack of practical information for everyday life issues [13]. The lack of such information can lead to feelings of resignation and fear among patients and their caregivers [13].

To meet these needs for PLS on the current management guidelines on MPS VI and MPS IVA, it was decided to use a visually appealing approach in the form of an infographic. An infographic is a visual diagram often used to convey complex information in a way that can be quickly and easily understood [20]. The use of infographics as a tool for summarizing and disseminating medical literature online and in print is increasing in popularity. Indeed, infographic-style summaries have been associated with higher reader preference and lower perceived mental effort during summary review compared with text-only summaries when assessed in disease states including psoriatic arthritis and multiple sclerosis [21, 22]. In addition to the chosen medium of infographics, we developed a visual design theme utilizing a “superhero” motif. This design was proposed by the authors and contributors in the hope it may help engage and empower an adolescent to young adult audience.

Access to information that allows a patient and their caregiver to understand the cause of their rare disease, its symptoms, and impact is an important requirement for being able to cope with the disease in everyday life [13, 23]. Patients who have access to understandable information around the treatment of their condition may also be in a better position to educate any non-expert HCPs they encounter, and thereby raise awareness of independently developed guidelines. As well as increasing the involvement of patients and caregivers in clinical care, engaging patients, caregivers, and patient advocacy groups in research can also play a key role in addressing evidence gaps for the management of rare diseases [11]. Furthermore, as a lack of information contributes to a lower than desired participation in clinical trials and other research into rare diseases [24], using PLS to increase awareness of relevant research projects being planned or conducted has the potential to increase the number of patients willing to participate.

Potential limitations associated with the development of these PLS for the MPS IVA and MPS VI guidelines should be acknowledged. Loss of nuance and/or precise detail may result from simplifying extensive technical texts. We hoped to minimize this issue by relying on an iterative approach to obtain alignment across the specialist members of our team and take in feedback at each step. Producing English language PLS may also be considered insufficient for a global patient audience from multiple cultural backgrounds and who may be non-fluent in English. However, our team included specialists based in a range of different countries aiming to reflect the global patient experience. Our use of plain language and clear images may also serve to lessen the issue of understandability for individuals who read English as a second or third language. However, the production of translated versions would represent an ultimate goal to obtain maximum audience reach, an approach that may be pursued in future with the materials presented here. While the infographics were developed with feedback from senior members of patient advocacy organizations, their real-world effectiveness in the primary target audience of patients has not been evaluated. A potentially valuable avenue of further research would be to gain additional feedback following the dissemination of these materials to patients and their caregivers and identify areas for improvement.

## Conclusions

In conclusion, PLS have a central role to play in ensuring that the latest scientific research reaches wider audiences and we recommend that they become standard practice for pivotal publications such as disease management guidelines. The use of PLS to clearly convey the complex information included in such guidelines will ensure that patients and their caregivers are well informed about their disease and able to participate in decisions around their care in a more meaningful and constructive way.

## Data Availability

Not applicable.

## Abbreviations

ARSB: *N*-Acetyl-galactosamine-4-sulfatase (arylsulfatase B)
CS: Chondroitin sulfate
CT: Computed tomography
ECG: Electrocardiogram
ENT: Ear-Nose-Throat
ERT: Enzyme replacement therapy
GA: General anesthetics
GAG: Glycosaminoglycan
GALNS: *N*-Acetyl-galactosamine-6-sulfatase
HCPs: Healthcare professionals
HSCT: Hematopoietic stem cell therapy
IV: Intravenous
KS: Keratan sulfate
MDT: Multidisciplinary team
MPS: Mucopolysaccharidosis
MRI: Magnetic resonance imaging
PLS: Plain language summaries
TIVAD: Totally Implantable Venous Access Device
